# Multiple Renal Cyst Development but Not Situs Abnormalities in Transgenic RNAi Mice against Inv::GFP Rescue Gene

**DOI:** 10.1371/journal.pone.0089652

**Published:** 2014-02-24

**Authors:** Yuki Kamijho, Yayoi Shiozaki, Eiki Sakurai, Kazunori Hanaoka, Daisuke Watanabe

**Affiliations:** Laboratory of Molecular Embryology, Department of Bioscience, Kitasato University School of Science, Sagamihara, Kanagawa, Japan; Institut National de la Santé et de la Recherche Médicale, France

## Abstract

In this study we generated RNA interference (RNAi)-mediated gene knockdown transgenic mice (transgenic RNAi mice) against the functional Inv gene. Inv mutant mice show consistently reversed internal organs (situs inversus), multiple renal cysts and neonatal lethality. The Inv::GFP-rescue mice, which introduced the Inv::GFP fusion gene, can rescue inv mutant mice phenotypes. This indicates that the Inv::GFP gene is functional *in vivo*. To analyze the physiological functions of the Inv gene, and to demonstrate the availability of transgenic RNAi mice, we introduced a short hairpin RNA expression vector against GFP mRNA into Inv::GFP*-*rescue mice and analyzed the gene silencing effects and Inv functions by examining phenotypes. Transgenic RNAi mice with the Inv::GFP-rescue gene (Inv-KD mice) down-regulated Inv::GFP fusion protein and showed hypomorphic phenotypes of inv mutant mice, such as renal cyst development, but not situs abnormalities or postnatal lethality. This indicates that shRNAi-mediated gene silencing systems that target the tag sequence of the fusion gene work properly *in vivo*, and suggests that a relatively high level of Inv protein is required for kidney development in contrast to left/right axis determination. Inv::GFP protein was significantly down-regulated in the germ cells of Inv-KD mice testis compared with somatic cells, suggesting the existence of a testicular germ cell-specific enhanced RNAi system that regulates germ cell development. The Inv-KD mouse is useful for studying Inv gene functions in adult tissue that are unable to be analyzed in inv mutant mice showing postnatal lethality. In addition, the shRNA-based gene silencing system against the tag sequence of the fusion gene can be utilized as a new technique to regulate gene expression in either *in vitro* or *in vivo* experiments.

## Introduction

RNA interference (RNAi) is an evolutionarily conserved mechanism for sequence-specific post-transcriptional gene silencing among species from various kingdoms. It has been shown that the introduction of short duplexes of synthetic 21–23 nt RNAs (siRNA) into mammalian cells has a gene-specific silencing function. However, transfected synthetic siRNA works for only a few days in mammalian cells. Therefore, another technical approach is required for continuous gene silencing.

Vector-based systems using RNA polymerase III promoters that stably produce siRNA from short hairpin RNA (shRNA) molecules have been established [1]–[5]. The production of RNAi-mediated gene knockdown transgenic mice (transgenic RNAi mice) has been demonstrated with GFP transgenic mice, which introduced a shRNA expression vector against GFP mRNA (pGtoR) [6], [7]. However, there are still few reports discussing the production of transgenic RNAi mice that show specific phenotypes caused by the down-regulation of the functional endogenous gene [8]–[11].

Recently, some transgenic RNAi mice that target endogenous functional genes were produced through knockdown ES cell lines that introduced a shRNA expression vector during *in vitro* experiments [12]–[14]. Recent new technical approaches, such as lenti virus-mediated transfection or site direct integration of the RNAi vector, that result in the production of transgenic RNAi mice with predicted phenotypes have been reported [15]–. Unfortunately, a lot of time and expense is required for ES cell screening and production of transgenic mouse lines from ES cells. In addition, the technical variability and complexity, as well as the unstable phenotypes resulting from RNAi mice, have limited their widespread use. Therefore, a technical approach able to produce transgenic RNAi mice easily, efficiently, and with resulting stability in the phenotype is required.

The Inversin gene (Inv) was identified as a protein of 1,062 amino acids containing an ankyrin repeat and IQ motifs deleted in inv mutant mice. Inv mutant mice show consistently reversed internal organs (situs inversus), multiple renal cysts (polycystic kidney), jaundice and neonatal lethality. This suggests that the Inv gene may play a very important role in left/right axis determination and kidney development during ontogenesis [22], [23].

In inv mutant mouse embryos, TGF beta family gene lefty and nodal, which are specifically expressed at the left side of the lateral plate mesoderm in the wild-type embryo, show a conversion in expression to the right side. This indicates that Inv may function in left/right axis determination at the very initiation step of mouse embryogenesis [24]–[26].

Recently, the INV gene in humans has been identified as the gene responsible for nephronophthisis type 2 (NPHP2), which shows a similar phenotype to inv mutant mice, such as kidney enlargement and cyst formation outside the medullary region with and without situs inversus [27]. Despite these findings, it is still unclear how the inv mutation induces left/right axis inversion and renal diseases because the physiological functions of the Inv gene have not yet been characterized. To characterize the physiological functions of Inv protein, we examined a transgenic rescue experiment.

The Inv::GFP-rescue mouse that introduced the Inv::GFP fusion gene into *inv* mutant mice (*inv/inv*, *Inv::GFP*) can rescue inv mutant mice phenotypes and does not show postnatal lethality or any developmental defects, such as situs inversus, renal cyst development or jaundice. This indicates that Inv::GFP fusion protein is functional *in vivo.* Furthermore, 9+0 type cilia-specific localization of Inv protein was demonstrated by ciliated tissues obtained from Inv::GFP-rescue mice by chasing GFP fluorescence expression in the cell [28], [29].

Inv protein is mainly observed in the transitional zone of cilia [30], [31]. Recently, it was suggested that cilia-localized Inv protein has a function in Wnt signal transduction [32], [33]. However, it is still unclear how Inv protein functions in 9+0 type cilia and regulates left/right axis determination and kidney development.

Inv protein is not only expressed in the mouse embryonic node or the renal tubules of the kidney but is ubiquitously expressed in whole embryonic cells and adult tissues [22], [23], [28]. Inv protein may have other functions in adult tissues containing 9+0 type cilia. However, early postnatal death of inv mutant mice makes it difficult to analyze Inv protein functions in adult tissues [34].

In this study we hypothesized that shRNA against GFP mRNA can degrade various GFP fusion mRNAs. To test this hypothesis we generated transgenic RNAi mice that targeted the Inv::GFP rescue gene (Inv-KD mice) by introducing the shRNA expression vector against GFP mRNA into Inv::GFP-rescue mice. We then monitored the gene’s knockdown effects by evaluating Inv-KD mice phenotypes caused by the down-regulation of the functional Inv::GFP-rescue gene. As expected, Inv-KD mice specifically down-regulated the Inv::GFP-rescue gene and showed hypomorphic phenotypes of inv mutant mice. This shRNA-mediated gene silencing system that targets the tag sequence of the fusion gene could be used for the regulation of functional transgenes that contain various tag genes for *in vivo* and *in vitro* experiments. Furthermore, Inv-KD mice that show renal cyst development but not postnatal lethality can be utilized to study Inv protein functions in adult tissue, as this is unable to be analyzed in inv mutant mice showing postnatal lethality.

## Materials and Methods

### Generation of Transgenic RNAi Mice

Vector DNA that expressed shRNA against GFP mRNA below the H1 promoter and HcRed mRNA below the CAG promoter (*pGtoR*) was kindly provided by Dr. M. Okabe [6]. RNAi-mediated gene knockdown mice were produced by the pronuclear injection of SalI and Bam HI digested pGtoR vector DNA into fertilized eggs obtained from the mating of *Inv::GFP inv/+* FVB mice [28]. The presence of the pGtoR and Inv::GFP transgenes was verified by the expression of HcRed and GFP fluorescence in newborn mice tails, which was observed with a fluorescent stereomicroscope (Lumar, Carl Zeiss, Germany).

For pGtoR, seven independent lines of transgenic RNAi mice were obtained. From the transgenic lines produced, those expressing HcRed fluorescence at a relatively high level (#1, #4 and #7) were used in the study. Founder RNAi mice were *Inv/+*or *+/+* and *Inv::GFP/+*.

### Western Blotting

Mouse embryos (e 18.5) and adult testis were lysed in cell lysis buffer containing 50 mM Tris HCl, 1% NP-40, 0.1% SDS, 0.5% Deoxycholate, 150 mM NaCl, 5 mM EDTA and 1 mM PMSF. Lysates containing 20 µg of protein were loaded onto 10% SDS-polyacrylamide gels and stained with Coomassie Brilliant Blue or transferred to nitrocellulose membranes. Polyclonal anti-GFP antibodies (MBL) and horseradish peroxidase-conjugated goat anti-rabbit antibodies (Jackson) were used as the primary and secondary antibodies, respectively. Immune complexes were detected using ECL Western Blot Detection System (Amersham).

### Histological Analysis

Kidney samples obtained from Inv-KD mice (*inv/inv*, *Inv::GFP*, *pGtoR*) and control Inv::GFP-rescue mice (*inv/inv*, *Inv::GFP*) were fixed in Bouin’s solution, dehydrated and embedded in paraffin wax. Serial sections (8 µm) were prepared and stained with hematoxylin and eosin (HE) according to standard procedures. To measure the degree of cyst formation in each mouse, the average number of cysts per a single kidney cross section was calculated using HE stained sections size dependently (diameter 400–800 µm and over 800 µm).

We analyzed 22 Inv-KD mouse kidneys and eight control Inv::GFP-rescue mouse kidneys obtained from12-month-old mice and from 1–1.5 month-old Inv::GFP-rescue mice or Inv-KD mice. The average number of cysts per single kidney cross section and the standard error of the mean from each transgenic RNAi mouse line were analyzed. Total areas of cystic tubules obtained from 12-month-old Inv-KD and Inv::GFP-rescue mouse kidney sections were analyzed with NIH imageJ software. The average percentage of cyst area per kidney cross section and the standard error of the mean were also analyzed. Statistically significant differences between two transgenic mice were determined using Student’s *t-*tests.

The experimental protocol was approved by the Institutional Animal Care and Use Committee of Kitasato University (permit number SA1117). Care was taken to minimize the number of animals used, and to reduce the amount of pain and suffering as much as possible.

### Immunohistochemistry

Freshly isolated mouse tissue was frozen in OCT compound (Tissue-Tec) and cryosectioned (8 µm). After fixation with 2% paraformaldehyde, the sections were incubated with rabbit polyclonal antibodies to GFP (MBL), mouse monoclonal antibodies to acetylated tubulin (Sigma), rat monoclonal antibodies to calmegin, rat monoclonal antibodies to PECAM-1 (Pharmingen) or peanut agglutinin (PNA)-FITC (Vector) [35]–[37]. Antibodies were diluted in phosphate-buffered saline containing 10% Blocking One (Nacalai Tesque) and 0.05% Tween 20. Immune complexes were detected with Alexa 568-conjugated goat antibodies to rabbit immunoglobulin G and Alexa 488-conjugated goat antibodies to mouse immunoglobulin G or Alexa 488-conjugated goat antibodies to rat immunoglobulin G (Molecular Probes). The sections were counterstained with Hoechst 33258 (Sigma).

## Results

### Generation of Transgenic RNAi Mice against Functional Inv::GFP Rescue Gene

**Figure 1:**
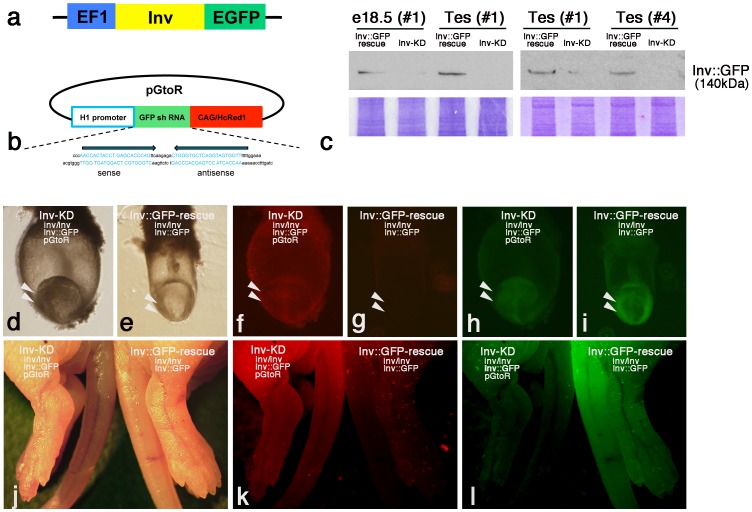
shRNA-based gene silencing of the Inv::GFP fusion gene. a: Construct of the Inv::GFP fusion gene introduced into Inv::GFP-rescue mice. EGFP coding cDNA was fused to the C terminus of Inv coding cDNA and inserted downstream of the EF1αpromoter [28]. b: Construct of the pH1/siRNAEGFP-CAG/HcRed1 (pGtoR) transgene. Two oligonucleotides containing sense and antisense 21 nt sequences from the EGFP coding region and a spacer sequence that provided a loop structure were inserted downstream of the H1 promoter [6]. HcRed1 coding cDNA was inserted into the vector downstream of the CAG promoter. c: Western blot analysis of Inv::GFP protein of embryonic day 18.5 (e18.5) embryos and adult testis. The lysates of e18.5 embryos (#1 line) and adult testis (#1 and #4 line) of pGtoR-integrated Inv-KD mice (*inv/inv*, *Inv::GFP*, *pGtoR*) and control litter mate Inv::GFP-rescue mice (*inv/inv*, *inv::GFP*) were analyzed by western blotting with anti-GFP antibodies. A band of approximately 140 kDa of Inv::GFP fusion protein was detected in Inv::GFP-rescue mice, but was specifically down-regulated in the pGtoR integrated Inv-KD mice embryos and testis. Accuracy of sample loading is indicated by the Coomassie-stained gel shown below. Bright field (d, e and j) and Hc-Red (f, g and k) or GFP (h, i and l) fluorescent observation of e8.0 embryos (d–i, arrowheads) and new bone (j–l) of Inv-KD mice (d, f, h and j–l, left) and control Inv::GFP-rescue mice (e, g, i and j–l, right) are shown. Hc-Red fluorescent was specifically expressed in Inv-KD mice but not control Inv::GFP-rescue mice (f, g and k). Inv::GFP fluorescent is specifically down-regulated in Inv-KD mice compared with Inv::GFP-rescue mice (h, i and l).

We hypothesized that shRNA that targeted for GFP mRNA can degenerate not only GFP mRNA but also GFP fusion mRNA. We attempted to demonstrate this possibility by generating RNAi-mediated gene knockdown transgenic mice (transgenic RNAi-mice) against the functional GFP rescue gene by using the transgenic Inv::GFP-rescue mouse lines. This rescues inv mutant mouse phenotypes through the expression of the functional Inv::GFP fusion gene (*inv/inv*, *Inv::GFP*) and the pGtoR vector, which continuously express shRNA against GFP mRNA (Fig. 1a and b).

The pGtoR vector expresses shRNA against GFP mRNA below the human H1 promoter and also the HcRed gene below the CAG promoter. This is designed to monitor the genomic integration of the transgene by the expression of red fluorescence (Fig. 1b) [6].

Using Inv::GFP-rescue mice as a target of the transgenic RNAi system makes it possible to monitor the gene knockdown effects *in vivo*. This is because it shows the apparent phenotypes of the inv mutant mice, such as situs inversus or renal cyst development, and depends on the degree of down-regulation of the functional Inv::GFP rescue gene.

Furthermore, Inv::GFP knockdown transgenic mice in the Inv::GFP-rescue mice genetic background (Inv-KD mice, *inv/inv*, *Inv::GFP*, *pGtoR*) can be used to analyze Inv gene functions in adult tissues. This cannot be analyzed in inv mutant mice showing postnatal lethality.

To analyze the shRNA-mediated gene knockdown effect against Inv::GFP fusion mRNA, we first transfected the pGtoR vector into fibroblast cell lines that continuously expressed Inv::GFP protein [28]. Inv::GFP protein was specifically down-regulated in fibroblast cells that express the pGtoR vector (data not shown). We then introduced the pGtoR vector into fertilized mice eggs obtained from *inv/+ Inv::GFP* transgenic mice.

Through injection of the pGtoR vector into fertilized eggs obtained from Inv::GFP-rescue mice, we were able to established seven independent transgenic RNAi mouse lines. These lines were verified by the existence of red fluorescence under fluorescent stereo microscopy observation (Fig. 1 k).

We selected three independent Inv-KD mouse lines that expressed relatively high levels of Hc-Red fluorescent (#1, #4 and #7) for use in further experiments.

In embryonic day 8.0 (e8.0) embryos, Inv::GFP fluorescent was specifically down-regulated in the embryonic region of Inv-KD mice (*inv/inv*, *Inv::GFP*, *pGtoR*) positive for Hc-Red fluorescent (Fig. 1d f and h, arrowhead) compared with Inv::GFP-rescue mice (*inv/inv*, *Inv::GFP*) (Fig. 1e, g and i).

We also observed the specific down-regulation of Inv::GFP fluorescent in new bone of Hc-Red-positive Inv-KD mice compared with Inv-rescue mice (Fig. 1j–l). The levels of Inv::GFP protein down-regulation were not stable and varied, even in litter mate transgenic mouse lines as observed by fluorescent stereomicroscopy. We did not observe a complete knockdown of Inv::GFP protein in this Inv-KD mouse line except for in testicular germ cells. By western blot analysis, we detected an approximately 140 KDa Inv::GFP fusion protein band from embryonic day 18.5 (e18.5) Inv::GFP-rescue mouse embryos and adult testis. In contrast, all three Inv-KD mouse lines examined (#1, #4 and #7) specifically down-regulated Inv::GFP protein and expressed less than 20% Inv::GFP protein compared with litter mate control Inv::GFP rescue mice (Fig. 1c, data not shown). These results were consistent with Inv::GFP fluorescent observations of Inv-KD mice.

Anatomical analysis of the neonatal Inv-KD mice did not show any lateral defects, including situs inversus, situs abnormalities, jaundice or renal cyst development. The Inv-KD mice did not show neonatal lethality and grew to fertile adulthood. However, they did show less viability and fertility at 12 months old compared with litter mate Inv::GFP rescue mice.

### Renal Cyst Development in Inv-KD Mice

Despite the specific down-regulation of Inv::GFP protein in the Inv-KD mouse embryos, we could not find any obvious phenotypes in neonatal Inv-KD mice.

**Figure 2:**
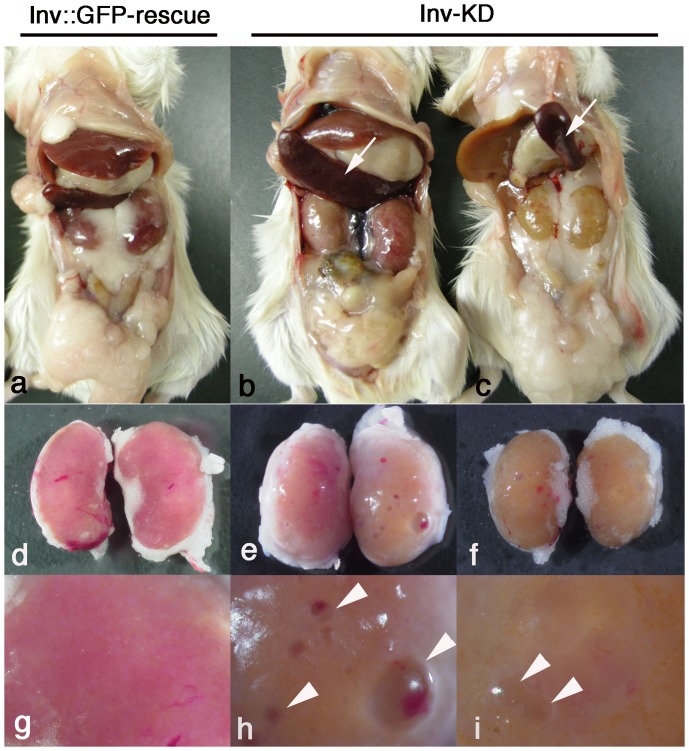
Anatomical analysis of adult Inv-KD mice. Anatomical analysis of pGtoR integrated adult (18 months old) Inv-KD mice (b and c) and control litter mate Inv::GFP-rescue mice (a). High magnification images of the kidney of (a–c) are shown in panels (d–f) and (g–i). Inv-KD mice developed fibrotic kidney with multiple cysts, but this was not observed in Inv::GFP-rescue mice. Highly developed multiple cysts were observed on the surface of Inv-KD mouse kidneys (h and i, arrowheads). Enlarged spleens were observed in adult Inv-KD mice (b and c, arrows).

We then we analyzed the internal organs of adult Inv-KD mice. Of the older Inv-KD mice (18 months old, six mice), we found apparent polycystic kidney development that showed a mildly enlarged and fibrotic kidney with apparent multiple cysts at high frequency (five mice) and splenomegaly (four mice) that were not observed in litter mate Inv::GFP-rescue mice (Fig. 2 a–i).

**Figure 3:**
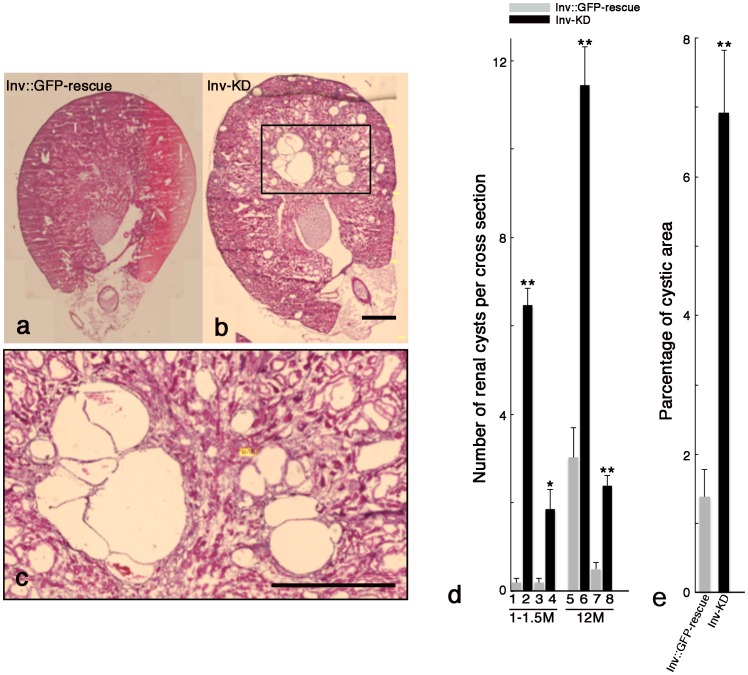
Multiple renal cyst development in Inv-KD mice. Cross sections prepared from adult (12 months old) Inv-KD mice (b) and control litter mate Inv::GFP-rescue mouse kidneys (a). High magnification image of the boxed region of (b) is shown in panel (c). Multiple renal cysts were observed in Inv-KD mice. The average number of cysts per single kidney cross section at 1–1.5 months old (lanes 1–4) or 12 months old (lanes 5–8) of kidneys in Inv-KD mice (lanes 2, 4, 6 and 8) and control litter mate Inv::GFP-rescue mice (lanes 1, 3, 5 and 7), diameter 400–800 µm (lanes 1, 2, 5 and 6) and over 800 µm (lanes 3, 4, 7 and 8) are shown (d). Average percentage of cyst area per single kidney cross section of adult (12 months old) Inv::GFP-rescue and Inv-KD mice are shown (e). Error bars represent S.E.M. (*) and (**) indicate Student’s t-test values <0.05 and <0.001, respectively. Scale bars: 1 mm.

We further analyzed the renal cyst development using HE stained kidney sections. Of the adult Inv-KD mice (12 months old, 22 mice), we could not find any incidence of splenomegaly, but histological analysis showed multiple cyst development in both the cortex and the medulla (Fig. 3b and c). The average cyst number per single kidney cross section was 11.4 for diameter 400–800 µm and 2.4 for diameter over 800 µm. Control Inv::GFP rescue mice showed 3.0 for diameter 400–800 µm and 0.5 for diameter over 800 µm (Fig. 3d, lanes 5–8).

In the 1–1.5 month-old Inv-KD mice, we did not observe fibrotic kidney or cyst formation on the kidney surface. However, developing small cysts were detected in both the medulla and cortex by histological analysis. The average number of cysts per single kidney cross section was 6.5 for diameter 400–800 µm and 1.9 for over 800 µm. Inv::GFP-rescue mice showed less than 0.2 cysts (Fig. 3d, lane 1–4). The average percentage of cyst area per single kidney cross section (12 months old, 22 mice) was 6.92% in Inv-KD mice and 1.39% in control Inv::GFP-rescue mice (Fig. 3e).

These results suggest that Inv-KD mice developed multiple renal cysts after birth and the enlarged and fibrotic kidney formed in adult Inv-KD mice with aging. These results were not transgenic Inv-KD-mice line-specific because similar phenotypes, such as fibrosis and enlargement of the kidney and multiple renal cyst development, were also observed in two other Inv-KD mouse lines (data not shown).

To analyze the cell types that down-regulate the functional Inv::GFP protein of the Inv-KD mouse kidneys that showed renal cyst development, we examined the expression of Inv::GFP protein by immunohistochemical staining.

**Figure 4:**
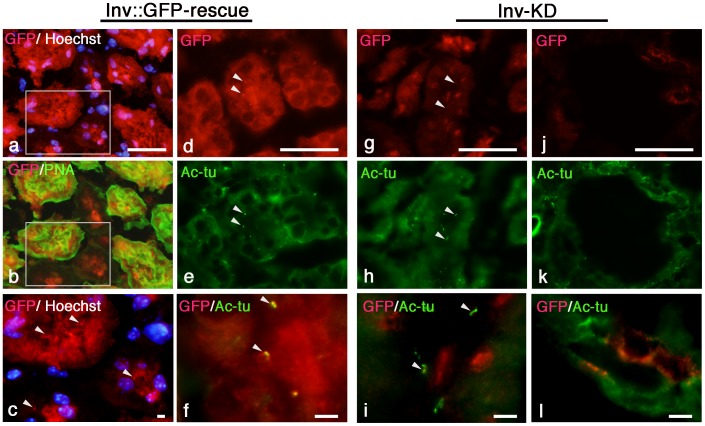
Down-regulation of Inv::GFP protein in Inv-KD mouse kidneys. Cross sections prepared from adult (12 month-old) kidneys of Inv-KD mice (g–l) and control Inv::GFP-rescue mice (a–f) were double-stained with anti-GFP antibodies (a–c, red) and PNA-FITC (b, green) or anti-GFP (d, g and j, red) and anti-acetylated tubulin antibodies (e, h and k, green). Nuclei were counterstained with Hoechst dye (a and c). High magnification image of the boxed region (a and b) are shown in panel (c). Merged and high magnification image of (de, gh and jk) are shown in panel (f, i and l) respectively. Inv::GFP protein was detected in proximal (PNA-negative) and distal (PNA-positive) tubules of Inv::GFP-rescue mice (a and b). Localization of Inv::GFP protein was detected in the cilia of both proximal and distal tubules (c, arrowhead).Inv::GFP protein was specifically down-regulated in the renal tubules of Inv-KD mice (g and j) compared with Inv::GFP-rescue mice (a and d). Inv::GFP fusion proteins were localized in the proximal region of anti-acetylated tubulin positive cilia of the renal tubules of Inv::GFP-rescue mice (d–f, arrowheads), but were down-regulated in Inv-KD mice (g–i, arrowheads). Inv::GFP protein was significantly down-regulated in the cystic tubules (j–l) compared with the non-cystic tubules (g–i). Images were taken at 10×40 (a and b) and 10×60 (d, e, g, h, j and k) magnification. Scale bars: 50 µm (a, d, g and j); 5 µm (c, f, i and l).

In the Inv::GFP-rescue mice kidneys, Inv::GFP protein was detected in the cells and cilia of PNA-positive distal tubules and PNA-negative proximal tubules (Fig. 4a, b and c, arrowhead). Inv::GFP protein was detected in all renal tubules including collecting duct cells of renal papilla (data not shown). Inv::GFP protein was specifically down-regulated in Inv-KD mice (Fig. 4g and j) compared with Inv::GFP-rescue mice (Fig. 4a and d). Double staining with anti-acetylated tubulin antibodies (which specifically recognize cilia of cells) and anti-GFP antibodies showed specific localization of Inv::GFP protein in the proximal region of cilia of the renal tubules cells (Fig. 4d–f, arrowhead), but cilia-localized Inv::GFP protein was down-regulated in Inv-KD mice compared with Inv::GFP-rescue mice (Fig. 4g–i, arrowhead). In Inv-KD mice, Inv::GFP protein was significantly down-regulated in the cells of the cystic tubules (Fig. 4j–l) compared with the non-cystic tubules (Fig. 4g–i).

### Germ Cell-specific Elimination of Inv::GFP Protein in Inv-KD Mouse Testis

**Figure 5:**
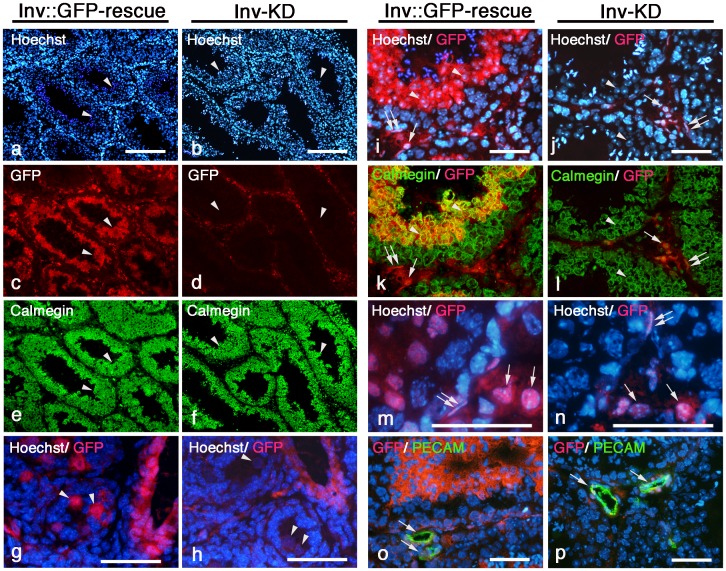
Testicular germ cell-specific elimination of Inv::GFP protein in Inv-KD mice. Testicular sections prepared from adult (a–f and i–p) or postnatal (3 day) (g and h) Inv-KD mice (b, d, f, h, j, l, n and p) or control Inv::GFP-rescue mice (a, c, e, g, i, k, m and o) were stained with anti-GFP antibodies (g, h, m and n, red) or double-stained with anti-GFP antibodies (c, d, i–l, o and p, red) and testicular germ cell-specific anti-calmegin antibodies (e, f, k and l, green) or vascular endothelial cell-specific PECAM-1 antibodies (o and p, green). Hoechst-stained images are shown to identify the cell types of the testis (a, b, g–i and m–p, blue). Inv::GFP protein was mainly observed in the spermatid cells of testicular germ cells (a, c, e, i and k, arrowhead) and somatic cells, such as connective tissue cells and Leydig cells (m, double arrows and arrow) and vascular endothelial cells (o, arrow) in Inv::GFP-rescue mice. Inv::GFP protein was specifically eliminated in the germ cells of Inv-KD mice (b, d, f, j, and l), and was down-regulated in connective tissue and Leydig cells (n, double arrow and arrow) and vascular endothelial cells (p, arrow). In Inv-KD mice, Inv::GFP protein was eliminated in anti-calmegin antibody-positive germ cells in adult testis, and in spermatogonia cells in postnatal mice (day 3) testis (h, arrowhead) but not in Inv::GFP-rescue mice (g, arrowhead). Scale bars: 200 µm (a and b); 50 µm (g−j and m–p).

To analyze the cell types that specifically down-regulate Inv::GFP protein in the testis, we analyzed adult Inv-KD mouse testis by immunohistochemical staining. In adult Inv::GFP-rescue mouse testis, Inv::GFP fusion protein was mainly observed in anti-calmegin antibody-positive spermatid cells and also in calmegin-negative non-germ cells, such as Leydig cells, and connective tissue cells consisting of seminiferous tubule and anti-PECAM-1 antibody-positive vascular endothelial cells (Fig. 5a, c, e, i, k, m and o). Inv::GFP protein was not detected in germ cells from spermatogonia to spermatocyte cells in adult Inv::GFP-rescue mice (Fig. 5i and k).

In contrast to Inv::GFP-rescue mice, Inv::GFP protein was specifically eliminated to undetectable levels in spermatid cells of Inv-KD mouse testis (Fig. 5d, j and l, arrowhead). However, testicular somatic cells, such as Leydig cells, and connective tissue cells, still expressed Inv::GFP protein and showed down-regulation of Inv::GFP protein as well as other somatic cells, such as renal tubule cells of the kidney (Fig. 5b, d, f, j, l, n and p). This testicular germ cell-specific elimination of Inv::GFP fusion protein was not Inv-KD mouse-line specific. All three Inv-KD mouse lines (#1, #4 and #7) showed similar results (data not shown). We were unable to analyze the elimination of Inv::GFP protein in spermatogonia cells (which localize to the basal layer of the seminiferous tubule) because of the low expression of Inv::GFP protein in adult Inv::GFP-rescue mice (Fig. 5i).

To analyze RNAi activity in spermatogonia cells, we analyzed postnatal mouse testis (day 3) that contained only spermatogonia cells as the germ cell. In the postnatal Inv::GFP-rescue mice testis, Inv::GFP protein was detected in spermatogonia cells as well as the non-germ cells including Leydig cells, connective tissue cells and vascular cells (Fig. 5g). In contrast, Inv::GFP protein was specifically eliminated in spermatogonia cells of the postnatal Inv-KD mice testis (Fig. 5h, arrowhead) compared with other somatic cells that showed the down-regulation of Inv::GFP protein (Fig. 5h).

The results indicate that RNAi-mediated gene silencing activities are specifically enhanced in the testicular germ cells compared with somatic cells.

## Discussion

### Generation of Transgenic RNAi Mice against Inv::GFP Rescue Gene

We hypothesized that continuous shRNA expression that targets the tag sequence of fusion gene mRNA can down-regulate fusion gene expression. We demonstrated this through the production of transgenic RNAi mice by using Inv::GFP-rescue mice. This rescues the inv mutant mouse phenotypes by expression of a functional Inv::GFP fusion gene and a vector-based RNAi system that continuously expresses shRNA against GFP mRNA and HcRed genes (pGtoR).

Expression of the pGtoR vector, which was introduced into Inv::GFP-rescue mice, specifically down-regulated Inv::GFP fusion gene expression *in vivo*. Transgenic RNAi mice showed hypomorphic phenotypes of inv mutant mice, such as renal cyst development in an age-dependent manner.

In our study, we obtained seven independent transgenic RNAi mouse lines through the injection of the pGtoR vector into approximately 200 fertilized eggs. All three of the transgenic RNAi mouse lines that were examined in this study consistently showed similar hypomorphic phenotypes of inv mutant mice.

By using the pGtoR vector, we can easily identify transgenic RNAi mice expressing shRNA by monitoring red fluorescence in the skin immediately after birth. This vector system will be very useful to detect transgenic RNAi mice or for the maintenance of mouse lines; and is perhaps more effective than genomic DNA analysis extracted from the tails of mice.

Recently, transgenic RNAi mice were produced thorough knockdown ES cell lines. Unfortunately, a great deal of time and expense is required for ES cell screening and production of transgenic mouse lines from ES cells. However, as this study describes, transgenic RNAi mouse production can now easily be accomplished through the direct injection of a RNAi vector into the fertilized egg. Through this novel shRNA vector system, the direct injection of a shRNA expression vector into a fertilized eggs can efficiently produce transgenic RNAi mice by using the proper target sequence as a shRNA expression vector, even if the sequence may target part of the tag sequence of the fusion gene. This RNAi system is not only applicable for gene knockdown analysis of transgenic mouse lines that are rescued by GFP fusion genes, but it is also applicable for gene knockdown analysis of other fusion genes containing fluorescent genes or tag genes such as myc, GST or Beta galactosidase in either *in vivo* or *in vitro* experiments.

This novel RNAi system, which targets the tag sequence of the fusion gene, can easily and consistently down-regulate target gene expression when compared with the common RNAi system, which targets for the gene-specific coding sequence. This is because it down-regulates the tag-containing target gene expression without being designed to select for the novel target gene-specific RNAi sequence in order to work efficiently. Furthermore, by introducing multiple transgenes that contain identical or different sets of tag sequences, it is possible to down-regulate multiple genes at once by controlling the tag-specific shRNA expression in either *in vivo* or *in vitro* experiments.

### Inv Function in Adult Tissues

From the results of this experiment, we observed that Inv-KD mice do not show lateral defects but do develop multiple renal cysts in an age-dependent manner, and Inv::GFP protein is both down-regulated in e8.0 embryos, which include node cells, and the kidney. This suggests the existence of different levels of Inv protein is required for left/right axis determination during early embryogenesis and kidney development.

In Inv::GFP-rescue mice, lateral defects, including situs inversus or situs abnormalities, were not observed. Small cyst development was rarely identified in older adult mice (Fig. 3d, data not shown). Inv::GFP-rescue transgenic mice that express C-terminal truncated Inv protein in inv mutant genetic background (inv/inv, InvΔC::GFP) do not show any lateral defects or postnatal lethality. However, they did develop severe polycystic kidney within two months [28]. These results suggest a relatively high level of Inv gene activity is required for normal kidney development compared with that required for left/right axis determination during mouse embryogenesis.

Inv-KD mice do not show postnatal lethality. However, they do gradually develop polycystic kidney during postnatal development, which makes it possible to analyze the developmental processes of renal cysts. This model could therefore be utilized as an experimental disease mouse model for human patients caused by the mutation of the INV/NPHP2 gene.

Some older mice (over 12 months old) showed other phenotypes such as splenomegaly, corneal keratinization and spinal curvature (Fig. 2b and c, data not shown).

During immunohistochemical analysis of Inv::GFP protein in adult Inv::GFP-rescue mice we detected the specific localization of Inv::GFP protein in 9+0 type cilia, such as in the choroid plexus, the pituitary gland and ependymal cells of the mouse brain and also in the axoneme of photoreceptor cells [28]. This suggests that Inv may have a function, not only in left/right axis determination and kidney development, but also in photoreceptor cells, choroid plexus cells, ventricular cells and pituitary gland cells which contain 9+0 type cilia.

Inv-KD mice, which show hypomorphic phenotypes of inv mutant mice, will assist in studying the unknown functions of the Inv gene in many tissues that cannot readily be analyzed in inv homozygous mutant mice showing neonatal lethality.

### Enhanced RNAi Activities in Testicular Germ Cells

Immunohistochemical analysis of Inv-KD mouse testis showed that Inv::GFP protein was eliminated in testicular germ cells, from spermatogonia to spermatid cells, compared with somatic cell such as Leydig cells and connective tissues. The enhanced RNAi activity appears male gametogenesis-specific as we did not observe elimination of Inv::GFP protein in oocytes (data not shown). These results suggest the existence of testicular germ cell-specific enhanced RNAi systems, which are distinguished from somatic cells and female gametogenesis.

It has recently been reported that murine spermatogenic cells express numerous endo-siRNAs, which are likely to be derived from naturally occurring double-stranded RNA (dsRNA) precursors [38], and that piwi interacting RNA, which has an important role in retro transposon gene silencing with the piwi family gene, is specifically expressed in male germ cells [39]–[43].

During mouse spermatogenesis, testicular germ cells may require enhanced RNAi-mediated gene silencing systems to repress many genes that inhibit normal male germ cell development.

To address the testicular germ cell-specific enhanced RNAi systems and to find the critical stage of RNAi activity during spermatogenesis, we are now introducing the pGtoR vector into other GFP transgenic mouse lines that consistently express GFP and cytoskeletal fusion protein in the testis. The Inv-KD mice produced in this experiment will be useful for studying Inv gene functions in adult tissues that cannot be analyzed in inv homozygous mutant mice by neonatal lethality.

This shRNA-mediated gene knockdown system, which targets for the predicted tag sequence of the fusion gene, will be very useful for regulating exogenously introduced tag-containing genes in both *in vitro* and *in vivo* experiments.
